# Internet addiction disorder: Fact or Fad? Nosing into Nosology

**DOI:** 10.4103/0019-5545.43622

**Published:** 2008

**Authors:** G. Swaminath

**Affiliations:** Department of Psychiatry, Dr. B. R. Ambedkar Medical College, Kadugondanahalli, Bangalore - 560 045, Karnataka, India

## TRAPPED IN THE NET

The Indian Institute of Technology (IIT) at Powai, Mumbai, prides itself for having churned out brilliant professionals who led this country's surge into the global economy as a software powerhouse. For such an institution, the restrictions on Internet use imposed recently for students in its hostels, drip with irony. “Tripping the Web Fantastic” is off. The genesis of the decision was from the suicide of a student, growing attendance problems in morning classes, and a fear that IITians were turning into unsocialized zombies, ensnared as they were in the worldwide web.

“Discomgoogolation”, a neologistic term from the findings of a survey of British Internet users, is meant to refer to a distressing condition, characterized by stress and anxiety at not being able to access the Internet. These Internet users felt frustrated at not being able to go online, experienced stress, and showed changes in brain activity and blood pressure. The survey opined that 76% of Britons could not live without the Internet, with over half of the population using the web between one and four hours a day and 19% of people spending more time online than with their family in a week.[1]

Starting as a hypothetical disorder, perhaps originating from a satirical hoax,[2] pathological Internet use – Internet Addiction Disorder (IAD) – seems to have stood its ground and its votaries promote its inclusion in both the *International Classification of Diseases 11^th^ Edition* (ICD 11) and *Diagnostic and Statistical Manual V Edition* (DSM V). Goldberg, who specializes in treating people with difficult-to-manage mood disorders, fabricated and posted a list of IAD symptoms on PsyCom.Net.[2] He said it was intended to be a parody of the DSM-IV, the present Bible for the diagnosis and reimbursement for treatment of mental health disorders. He compounded the irony by starting an Internet addiction support group online, akin to holding an Alcoholics Anonymous meeting in the middle of a cocktail party.[2] Goldberg's spoof on society's fascination with its own addictive behaviors took on a cyberlife of its own, and news of this new disorder spread among netizens.

Across the board, mental health experts agree that the Internet has provided valuable service to people looking for support groups, treatment options, and other help. Web sites, newsgroups, and E-mail lists all are powerful resources for people to find the information and help they need.

## A CLASS OF ITS OWN?

However, this categorization of IAD in diagnostic manuals causes concern among most mental health professionals. Robins and Guze[3] followed by Feighner *et al*,[4] proposed formal criteria for establishing the validity of psychiatric diagnoses. These are: 1) clinical description (including symptom profiles, demographic characteristics, and typical precipitants), 2) laboratory studies (including psychological tests, and radiology and postmortem findings), 3) delimitation from other disorders (by means of exclusion criteria), 4) follow-up studies (including evidence of diagnostic stability), and 5) family studies. Of these criteria IAD at present fulfils only clinical description, for there is as yet paucity of delimitation from other disorders, and of lab, family, and follow-up studies. An overview of etiology, assessment, and treatment, of IAD has appeared in print[5] skirting the basic issue of its validity.

Jerald Block in an editorial in the American Journal of Psychiatry recommends the inclusion of IAD in DSM-V. He opines that conceptually, the diagnosis is a compulsive–impulsive spectrum disorder that involves online and/or offline computer usage and consists of at least three subtypes: excessive gaming, sexual preoccupations, and E-mail/text messaging. These variants share the following four components: 1) excessive use, often associated with a loss of sense of time or a neglect of basic drives, 2) withdrawal, including feelings of anger, tension, and/or depression when the computer is inaccessible, 3) tolerance, including the need for better computer equipment, more software, or more hours of use, and 4) negative repercussions, including arguments, lying, poor achievement, social isolation, and fatigue.[6]

In June 2007, the American Medical Association (AMA) declined to recommend to the American Psychiatric Association that they include IAD as a formal diagnosis in the 2012 edition of the DSM, and recommended further research. A better definition of “overuse”, and a way to differentiate an “Internet addiction” from obsession, self-medication for depression or other disorders, and compulsion were still wanting.

“Internet addicts” already fit under existing, legitimate diagnostic labels. For many patients, overuse or inappropriate use of the Internet is merely a manifestation of their disinhibition in mania, enhancing self esteem, social networking, and relief in depression, social phobia, and net compulsions such as shopping, pornography, or gambling in OCD, sexual disorders, impulse disorders, or gambling. IAD is compared to food addiction, in which patients overeat as a form of self-medication for depression, anxiety, etc., without actually being truly addicted to eating.[8,9]

In IAD the form (i.e. Internet) is to be distinguished from the content (i.e. online activity such as gaming/gambling, or sexual preoccupations). To continue with the example of gambling: pathological Internet gambling could be regarded as a subtype of pathological Internet use (the category is primarily defined by form – Internet – and subdivided on the basis of content – gambling), or it could be regarded as a subtype of pathological gambling (the category is primarily defined by content and subdivided on the basis of form). This distinction will have implications for etiology, treatment, and prognosis.[8]

A person addicted to substances displays a strong compulsion to seek out drug dealers, difficulty in controlling their drug-dealer-seeking behavior, and progressive neglect of alternative pleasures in favor of seeking out drug dealers: they thereby fulfil ICD-10 criteria for a dependence syndrome to drug dealers. But “drug-dealer addiction” does not exist because we recognize that the drug dealer is only the conduit for the addictive substance and not the addictive substance *per se*.[8] Internet addiction is defined as Internet use of more than thirty-eight hours per week. Computer professionals, businessmen, online lecturers, professionals, students using E-learning, and even those of us in journal publication, use the Internet at least forty hours each week increasing the risk of these being classified as Internet addicts.[9] All of this suggests not only poor delimitation of IAD from other disorders but also from legitimate usage.

Inclusion of a diagnosis in classification requires careful consideration of the research underlying the disorder. Other considerations such as the sociological must also be taken into account.[10] After a series of ten cardiopulmonary related deaths in Internet cafés, a game-related murder, and many children dropping out of school and work to spend time on computers, South Korea considers IAD as one of its most serious public health issues[6] and has taken proactive measures such as training counsellors to prevent and to manage the condition.

## FADS: FADE IN/FADE OUT

Every proposed new diagnosis carries with it the risk of making a false-positive diagnosis, that is, making a diagnosis when no established disorder is present. Hence, the advantages of including the diagnosis in the classificatory system (e.g., increased detection of a treatable disorder with eventual reduction in morbidity and cost to the patient, his or her family, and to society at large), are to be weighed against the risks of making a false-positive diagnosis (e.g., risk of stigmatization, cost, and possibly unnecessary treatment, etc.).[10] Some proponents believe that its inclusion would open the doors for private insurance companies to pay for IAD counseling. On the contrary, self-proclaimed sufferers are approaching courts for redress. In one recent American case (Pacenza v. IBM Corp.), the plaintiff argued that he was illegally terminated in violation of the “Americans with Disabilities Act” owing to his Internet addiction triggered by Vietnam war-related “Post-Traumatic Stress Disorder”.[11] The case is pending before the court in the Southern district of New York.

With every new revision of ICD and DSM the number of categories has increased exponentially. Differentiation between subcategories is not well defined leading to confusion. In an international comparison performed to evaluate the frequency and use of the ICD-10 psychiatric diagnoses to assist in future revision of the ICD-10, an attempt was made to know which diagnostic categories were either not used or were used possibly in an unspecific manner. There were 32 specific diagnostic categories on a four-character level which are not used at all and 121 which were used less frequently than 0.1% in inpatient and outpatient treatment.[12]

This suggests a much bloated classificatory system. Future additions without sufficient data could further inflate the system reducing its utility in practice. Though sufficient research data might over time validate IAD, at present it seems a fad illness. True, the Internet contributes to the answering of many questions, but “Internet addiction” as of now raises more questions than can be answered.
